# Advancements in high-resolution imaging of the iridocorneal angle

**DOI:** 10.3389/fopht.2023.1229670

**Published:** 2023-08-21

**Authors:** Matthew J. Keller, Thomas J. Gast, Brett J. King

**Affiliations:** School of Optometry, Indiana University, Bloomington, IN, United States

**Keywords:** high-resolution imaging, iridocorneal angle, gonioscopy, trabecular meshwork, glaucoma

## Abstract

High-resolution imaging methods of the iridocorneal angle (ICA) will lead to enhanced understanding of aqueous humor outflow mechanisms and a characterization of the trabecular meshwork (TM) morphology at the cellular level will help to better understand glaucoma mechanics (e.g., cellular level biomechanics of the particulate glaucomas). This information will translate into immense clinical value, leading to more informed and customized treatment selection, and improved monitoring of procedural interventions that lower intraocular pressure (IOP). Given ICA anatomy, imaging modalities that yield intrinsic optical sectioning or 3D imaging capability will be useful to aid in the visualization of TM layers. This minireview examines advancements in imaging the ICA in high-resolution.

## 1 Introduction

Glaucoma is a chronic progressive neurodegenerative disease characterized by damage to retinal ganglion cells and their axons with a correlating loss of visual field (1). While numerous studies have identified several risk factors associated with glaucoma, the only modifiable risk factor proven to be efficacious in large, randomized clinical trials is intraocular pressure (IOP). IOP is among the most consistently established risk factors and plays an important role in glaucomatous neuropathy. It has also been demonstrated that reducing IOP decreases the risk of field progression by several studies (2–4). Currently, glaucoma management involves seeking to lower IOP through various pharmacological or surgical methods.

The conventional outflow pathway for aqueous humor through the iridocorneal angle (ICA) is a highly important component in IOP regulation. In this pathway, aqueous humor produced by the ciliary body epithelium travels through the multi-layered trabecular meshwork (TM) into Schlemm’s canal (SC), then passes through collector channels into the episcleral veins, entering into the venous circulation (5). ICA pathology can cause increased outflow resistance along this pathway, which can subsequently cause elevated IOP. This pathology can occur in the form of angle closure glaucoma or secondary glaucomas where aqueous outflow facility is disrupted (or other secondary glaucomas like pigmentary, neovascular, uveitic, etc.) or in open angle glaucomas, where the increased outflow resistance mechanics are not always clear.

Imaging the anatomy of the ICA is of high value for clinicians who wish to assess and evaluate its structures, especially in the context of glaucoma. The diagnostic ability of an imaging system used to assess glaucoma relies heavily on the benefits of obtaining highly-detailed ICA information. Unfortunately, high resolution ICA imaging remains a challenge even with the current advancement in techniques and approaches that seek to address this problem. Resolving ICA structures such as TM, SC, and the scleral spur in high detail is inherently difficult due to the relatively less accessible anatomical location. Because the ICA lies posterior to the peripheral cornea, limbus, and anterior sclera, direct incident light sources are subject to near total internal reflection and cannot be used for imaging. While clinical ICA assessment *via* slit-lamp gonioscopy can circumvent this, this technique is subject to significant intrarater and interrater variability (6). These challenges, as well as the limited resolution of this technique, pose limits to patient care and clinical research (7).

An ideal ICA imaging system must overcome these limitations to provide valuable information to researchers and clinicians. Several potential mechanisms have been hypothesized to describe glaucomatous pathology at the ICA. Examples of these include but are not limited to: presence of cells and cellular debris between trabecular lamellae beams that can plug filtration, TM sclerosis with altered collagen/elastin and increased basal lamina material, and endothelial cell attenuation within SC (8–10). As this pathology occurs within ICA tissue at the micron-level scale, with studies suggesting that uveal TM collagen beams are on average 4 μm and corneoscleral TM pores are as small as 5 μm, an imaging modality that seeks to provide greater understanding of disease processes and treatment mechanics must provide high resolution (11). Specifically, this level of investigation warrants cellular to subcellular spatial resolution (< 5 μm) of these ICA microstructures with high contrast, both at the surface as well as in depth. This is in the particular interest of researchers hoping to identify new biomarkers, to guide future clinical devices that seek to visualize TM disorders and aid in the screening and classification of glaucoma. Here, recent techniques and approaches to obtain high-resolution images of the iridocorneal angle with different modalities are discussed in consideration to future application.

## 2 Advances in gonio-photography

Several commercial gonio-photographic systems designed to record and qualitatively assess the ICA have been developed over the years (12). These angle photography methods exist to address limitations of slit lamp gonioscopy, such as consistent, high-quality documentation for sequential analysis, and to promote a greater uptake of gonioscopy among physicians. While a simple slit-lamp mounted camera setup can be used to take photographs of the ICA through a clinical gonio-lens, image resolution and focus significantly limit the ability of this setup to provide clear, discernible images of ICA structures.

**Figure 1:**
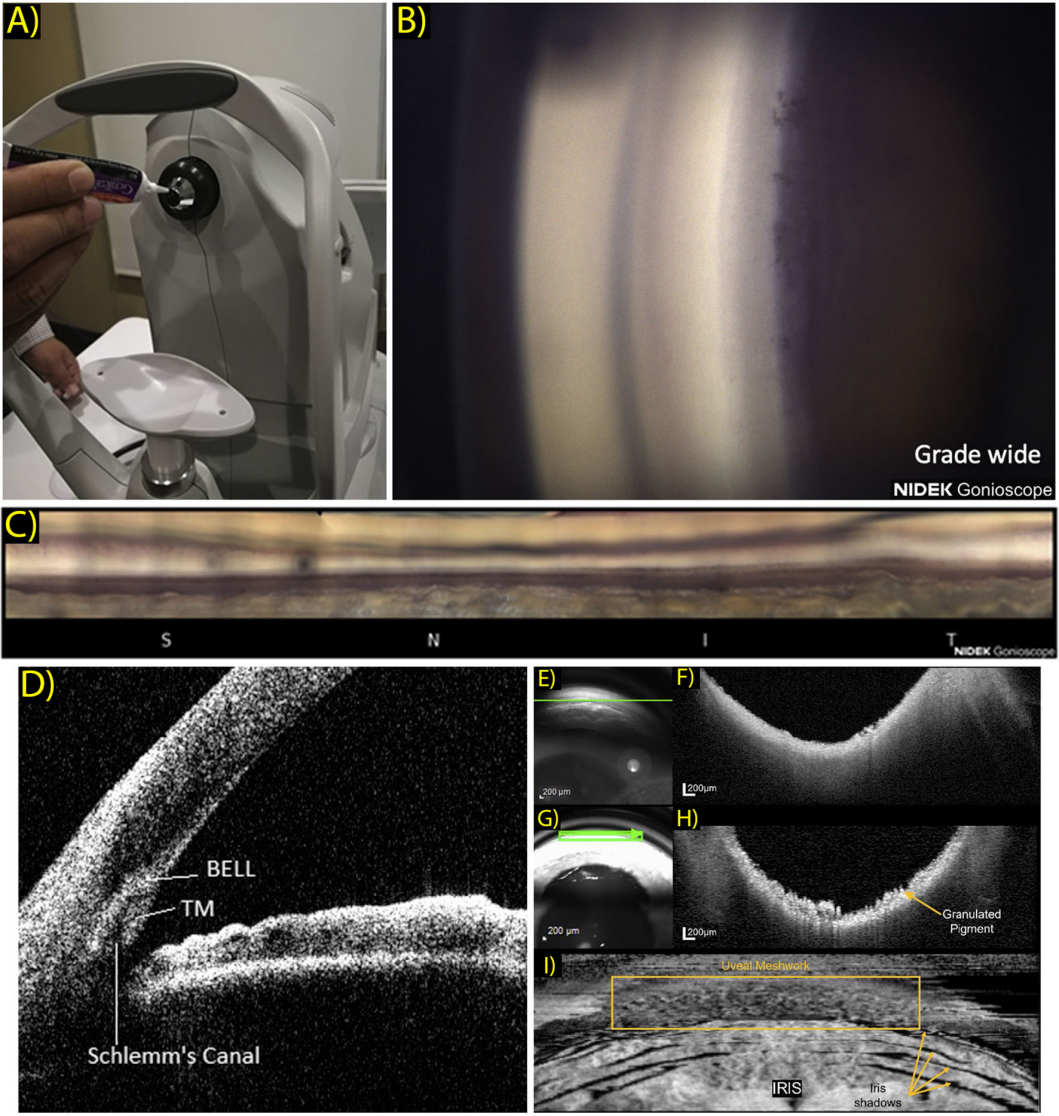
**(A)** Patient view of the NGS-1 gonioscope (NIDEK Co, Gamagori, Japan). Gel is applied onto the machine-mounted, 16-mirrored gonioscopic lens before contact is made with the subject’s eye for coupling to the camera system. (Ophthalmol Glaucoma. 2019 Jul 1;2(4):215–23. Originally published by and used with permission from Elsevier Inc.). **(B)** Example NGS-1 photograph. Photo shown displays an angle opening Scheie grade of wide, with all structures visible up to the iris root and its attachment to the ciliary body. (Ophthalmol Glaucoma. 2019 Jul 1;2(4):215–23. Originally published by and used with permission from Elsevier Inc.). **(C)** Linear “unrolled” display of 360-degree montaged NGS-1 photos. (Ophthalmol Glaucoma. 2019 Jul 1;2(4):215–23. Originally published by and used with permission from Elsevier Inc.). **(D)** Two-dimensional CASIA SS-1000 anterior segment OCT iridocorneal angle image with labeled landmarks: trabecular meshwork (TM), Schlemm’s canal, band of extracanalicular limbal lamina. (Ophthalmology. 2018 Jul 1;125(7):994–1002. Originally published by and used with permission from Elsevier Inc.). **(E–H)** Gonioscopy SLO **(E)** and OCT b-scan **(F)** oriented tangentially to the iridocorneal angle acquired in a normal subject and correspondingly in a subject with pigment dispersion syndrome **(G, H)**. Scan orientation is shown as a green arrow in the SLO image. (Biomed Opt Express. 2022 Sep 1;13(9):4652. Originally published by and used with permission from Optica Publishing Group.). **(I)** Transverse *enface* OCT gonioscopy image of the iridocorneal angle. This image was constructed from a dense OCT volume scan acquired along the anterior surface of the trabecular meshwork. Uveal meshwork can be seen and is demarcated by the yellow box, iris folds create shadowing of the OCT beam as indicated by the yellow arrows. (Biomed Opt Express. 2022 Sep 1;13(9):4652. Originally published by and used with permission from Optica Publishing Group).

The EyeCam (Clarity medical systems, Pleasanton, CA) is an early gonio-photography system design that saw some success in clinical ICA research. This is a handheld commercial device, born out of usage modifications to the RetCam, a similar handheld system designed to acquire widefield retinal images (13). Also in development is the GonioPEN, a compact, handheld gonio-photography system that aims to improve limitations that the EyeCam faced, such as ease of use and cost (14). More recently the Nidek GS-1 gonioscope (NIDEK, Gamagori, Japan) was released commercially and has been established as a fast, user-friendly instrument for ICA photographic documentation (15). The GS-1 is an automated gonioscope device featuring an attached 16-mirror faceted gonioscopic lens for 360° imaging, with a total image acquisition process that can take less than 60 seconds per eye (16). This lens is coupled to an anesthetized eye with gel in a sequence that is comparable to traditional slit lamp gonioscopy. This system is capable of automatic focus adjustment as images are acquired at different focal planes within each sector. These images are selected automatically by software that determines an assumed optimal TM focus, which can be reselected manually by the operator. The software’s output image can then be visualized as a montaged linear or circular image of the entire 360° ICA configuration (**Figures 1A–C**).

While automated gonioscopy imaging is certainly promising, usage reports have determined varying degrees of reported operator ease of use and patient toleration. In a 2019 pilot study exploring GS-1 gonioscopy, Shi et al. performed imaging on 84 eyes, including 58 eyes of patients diagnosed with glaucoma (17). Of the resulting images over a range of angles from wide-open to narrow angles, 8.33% were deemed ungradable due to poor quality, with good fixation and reasonable patient cooperation cited as requirements to attain successful gonio-photographs. This instrument has proven useful to document and assess the position of minimally invasive glaucoma surgeries (MIGS) (18, 19).

While contact-imaging does provide benefits of motion stability, patient toleration must be considered before claims can be made of the usefulness of any contact imaging device. Barbour-Hastie et al. recently examined GS-1 images and aimed to incorporate patient satisfaction into their assessment of overall device utility in a clinical setting (20). In a study involving 25 patients, most reported the GS-1 imaging procedure equivalent or preferred to slit lamp gonioscopy. Variable ease of use was reported from the clinician operators but was never graded as ‘difficult’. Overall, 90.4% of GS-1 images acquired in this study had discernible ICA structures. This is an improvement over a previous report from Teixiera et al. who used an earlier design of the GS-1 system and reported a structure identification rate of only 63.1% in their images, and only slight angle opening grader agreement (*k* - 0.09) with manual gonioscopy and low inter-rater agreement for angle closure detection in the images (21). While the upside of fast, documentable, high-quality gonioscopic photography is clear, these images can be difficult to obtain, and the acquisition process is still limited by patient discomfort and operator familiarity. Further limitations include the inability of these contact photograph devices to perform dynamic gonioscopy, as well as capture the full three-dimensional ICA anatomy (16).

The use of artificial intelligence (AI) to reliably aid image interpretation by the physician is of high value and is being explored. Deep learning algorithms used to identify and segment angle structure, as well as classify into open or closed status, have proven to be fast and accurate (22–24). Chiang et al. recently developed a convolutional neural network (CNN) algorithm to classify EyeCam ICA images to a closed or open status (24, 25). The proposed classification software performance was compared against human graders in detecting angle closure in EyeCam gonio-photographs and reported superior classification and repeatability performance (*k* - 0.823, AUC - 0.969) based on a single-grader label. In a different approach utilizing AI, Peroni et al. have reported multiple algorithms designed to segment ICA anatomy in GS-1 images. In a recent report a segmentation rate of up to 91% was achieved across GS-1 test data with their newest segmentation software (26, 27). Using these systems in conjunction with AI is a future direction that should benefit clinician interpretation of gonioscopy and gonio-photographs, but sufficient agreement must be established. Additionally, no commercial gonio-photography system has spatial resolution sufficient to resolve TM structure.

## 3 OCT based imaging

### 3.1 AS-OCT

Many studies investigating the anterior segment and ICA have utilized modern optical coherence tomography (OCT) methods to obtain cross sectional views of ocular tissue. Anterior segment OCT (AS-OCT) was conceptually demonstrated not long after OCT was invented and has since garnered interest to evolve and become systematically optimized for imaging the anterior eye. Currently AS-OCT is a well-established technique with applications including investigation of cornea and lens pathology and visualizing the risk of angle closure (28). The non-contact nature of AS-OCT is generally more attractive from the patient’s perspective and also eliminates potential problems from inducing unnatural stresses on the cornea and ICA. This can be especially problematic in studies probing TM stress or angle configuration (29). Measuring anterior chamber biometric parameters with AS-OCT has become an imaging standard in terms of both image metric quality and ease of use (30–32).

OCT techniques have been continuously developed and tailored to anterior segment imaging (33). One technique is the use of comparatively longer laser wavelengths than currently used with spectral domain ocular coherence tomography (SD-OCT). This has the advantage of maintaining high contrast and signal strength while imaging directly through the overlying multiply-scattering limbus and sclera. Early commercial AS-OCT devices were time-domain OCT systems that did use long wavelength light, but also had slow scan speeds. Commercial SD-OCT systems optimized for retinal imaging are capable of high imaging speeds and have better axial resolution, but use shorter wavelength light sources (~880nm) with shallow depth range into the anterior segment. New AS-OCT systems utilize swept source OCT (SS-OCT) over SD-OCT, which permits fast imaging speed with longer laser wavelength (~1300nm), optimized to maintain sensitivity for deeper tissue imaging. Differences in technical specifications of these OCT systems are outlined in **Table 1**.

**Table 1:** Comparison of commercial AS-OCT technical specifications.

Manufacturer	OCT System	Light Source	Axial Resolution (μm)	Lateral Resolution (μm)	Imaging Depth (mm)
Carl Zeiss Meditec	Visante	1310 nm SLD	18	60	6 mm
Carl Zeiss Meditec	Cirrus^1^	840 nm SLD	5	15	2 mm
Heidelberg Engineering	Spectralis^1^	820 nm SLD	7	20	2 mm
Tomey Corporation	CASIA SS-1000	1310 nm SS laser	10	30	6 mm
Tomey Corporation	CASIAII	1310 nm SS laser	< 10	30	13 mm
Topcon Corporation	Triton^1^	1050 nm SS laser	8	30	6 mm
Heidelberg Engineering	Anterion	1300 nm SS laser	< 10	30	14 mm

SLD, Superluminescent diode; SS, Swept source; ^1^OCT instruments designed for retinal imaging, anterior segment imaging performed via an accessory lens.

In 2018, a study by Crowell et al. sought to address and characterize features and anatomical landmarks that can be visualized with new AS-OCT technology (34). The group reviewed AS-OCT images obtained using a commercial SS-OCT, CASIA SS-1000 (Tomey, Nagoya, Japan). They presented a novel landmark which was coined as the band of extracanalicular limbal lamina (BELL), a feature consists of an avascular layering of collagenous tissues that is posterior to the TM and is adjacent to external SC (**Figure 1D**). Though clinical and pathological significance of the BELL is currently unclear, it has been hypothesized that this tissue provides structural support to the ICA, and may correlate with fibers observed when entering SC intraoperatively.

While successful, trans-tissue SS-OCT imaging can still be limited by light scattering. Crowell et al. reported visualization of TM and BELL in 73% and 93% of studied individuals, respectively, but identified SC in only 40% of people. A further attempt by Ueno et al. utilized a custom polarization-sensitive OCT (PS-OCT) technique to detect and identify TM and BELL at higher rates by removing birefringence artifacts (31). With PS-OCT, BELL was identified in 99.2% of cases and TM identified in 95.1%. Conversely, SC is poorly resolved with PS-OCT due to its luminal nature, with visibility in only one subject (0.3%). Other strategies to better image SC have included a custom high speed 1.66-MHz SS-OCT prototype system, demonstrated to successfully obtain full 3D reconstruction renderings of SC in two eyes but with a relatively coarse lateral resolution of 17.54 μm (35).

AS-OCT output of both cross-sectional 2D images or reconstructed 3D ICA images also allows for easy assessment of structures relative to each other, which has proven useful to aid diagnosis of angle closure glaucoma (36–38). Common parameters derived from AS-OCT images include anterior chamber depth and width, and lens parameters like vault and thickness. Many features describing the angle have been established as biomarkers for angle closure, such as angle opening distance at 500 μm from the scleral spur, trabeculo-iris space, and angle recess area (39–41).

Many studies have also explored the implementation of AI algorithms to reliably detect ICA pathology in AS-OCT images (42–44). Success has been demonstrated in AI capability to screen for angle closure risk and identify features such as iridocorneal apposition and PAS (45). Future directions of deep learning supported algorithms must be able to provide diagnostic aid across multiple ethnicities and classify ICA opening grade to identify risk of angle closure.

While the clinical value of current AS-OCT is clear, image resolution is still a limitation of this modality, and tissue resolution is not sufficient for investigation of trabecular biomechanics at the cellular level and therefore provides no clinically useful information with regard to open angle glaucoma. These images also struggle to identify angle neovascularization and level of pigment, which means gonioscopy remains a necessity for comprehensive evaluation of the ICA (46).

### 3.2 OCT gonioscopy

In addition to using OCT to image TM and ICA structures externally through the limbus, OCT based gonioscopy approaches have also been successfully reported. This concept utilizes a contact gonioscopy lens to image the ICA from inside the eye, circumventing total internal reflection. McNabb et al. described a customized OCT gonioscopy system they designed to obtain 360° ICA images (47). They constructed an SS-OCT system to be coupled with a curved 360° paraboloidal mirror gonio lens to image the entire ICA circumference with a single volume. While this method proved effective and could construct entire 360° ICA 3D volume renderings with relatively short acquisition time, the lateral resolution of this system was 24 μm, far too large for resolving the smaller trabecular structures.

Carmichael et al. reported a similar OCT gonioscopy technique, but with the use of a commercially available SD-OCT (Heidelberg Spectralis, Heidelberg Engineering, Heidelberg, Germany) and modified clinical goniolens (48). With this strategy, they improved system ease of use and reduced cost of specialization, and were able to obtain gonio OCT images of the ICA with an axial resolution calculated at 8.35 μm (**Figures 1E–I**). They displayed reconstructed enface images and were able to obtain representations of uveal meshwork in higher detail. This is an efficient strategy that can potentially permit cost-effective ICA imaging at an easier uptake rate.

### 3.3 Time-domain full-field OCT

While the previously mentioned OCT-based designs offer many benefits like ease of use and low-cost, the reported resolution of the CASIA SS-1000 is not suited for resolving cellular features at axial 10 μm and lateral 30 μm (33). Although gonioscopic OCT methods successfully obtained images of ICA with improved resolution, neither design was able to provide cellular level structural information of the ICA region. These methods also both employ the use of contact imaging which can limit patient comfort.

To address this, Mazlin et al. recently developed a novel multimodal anterior OCT system that combined time-domain full-field OCT (TD-FF-OCT) and SD-OCT. Full field OCT obtains high resolution en face images *via* simultaneous illumination of the entire scanning field. The use of TD-FF-OCT provides the benefit of local, cell-resolution images, which this group first reported for *in vivo* human cornea imaging in 2018 (49). Recent upgrades to this system incorporated benefits of an SD-OCT arm. This strategy yields cellular-resolution en face TD-FF-OCT images in the cornea, while simultaneously acquiring cross-sectional SD-OCT images with large field of view, so that real-time imaging position can be determined (50).

Recently, they were able to acquire *in vivo* human ICA images with a reported lateral resolution of 1.7 μm (51). Using this method to image a single subject, Mazlin et al. produced images of TM fibers and corneoscleral and uveal meshwork were able to be differentiated. Notably, their OCT based system corneal images can be acquired quickly, with alignment and acquisition reported to take only minutes. Alignment time for trabecular meshwork imaging, however, was said to be substantial.

## 4 Non-OCT based imaging

### 4.1 Two-photon microscopy

Two-photon(2P) microscopy is a popular imaging technique utilized to investigate biological tissue in many different scientific domains. Its use to image ex vivo and *in situ* human and animal ICA tissue has also been established to differentiate and characterize structure details (52–54). Photon emission signals from fluorophores are collected to form an image upon stimulation with excitation light. 2P microscopy provides many beneficial attributes considering its application to obtaining ICA images. In particular, tissue photo-absorption is restricted to the imaging plane, which permits deep laser penetration and affords intrinsic optical sectioning.

**Figure 2:**
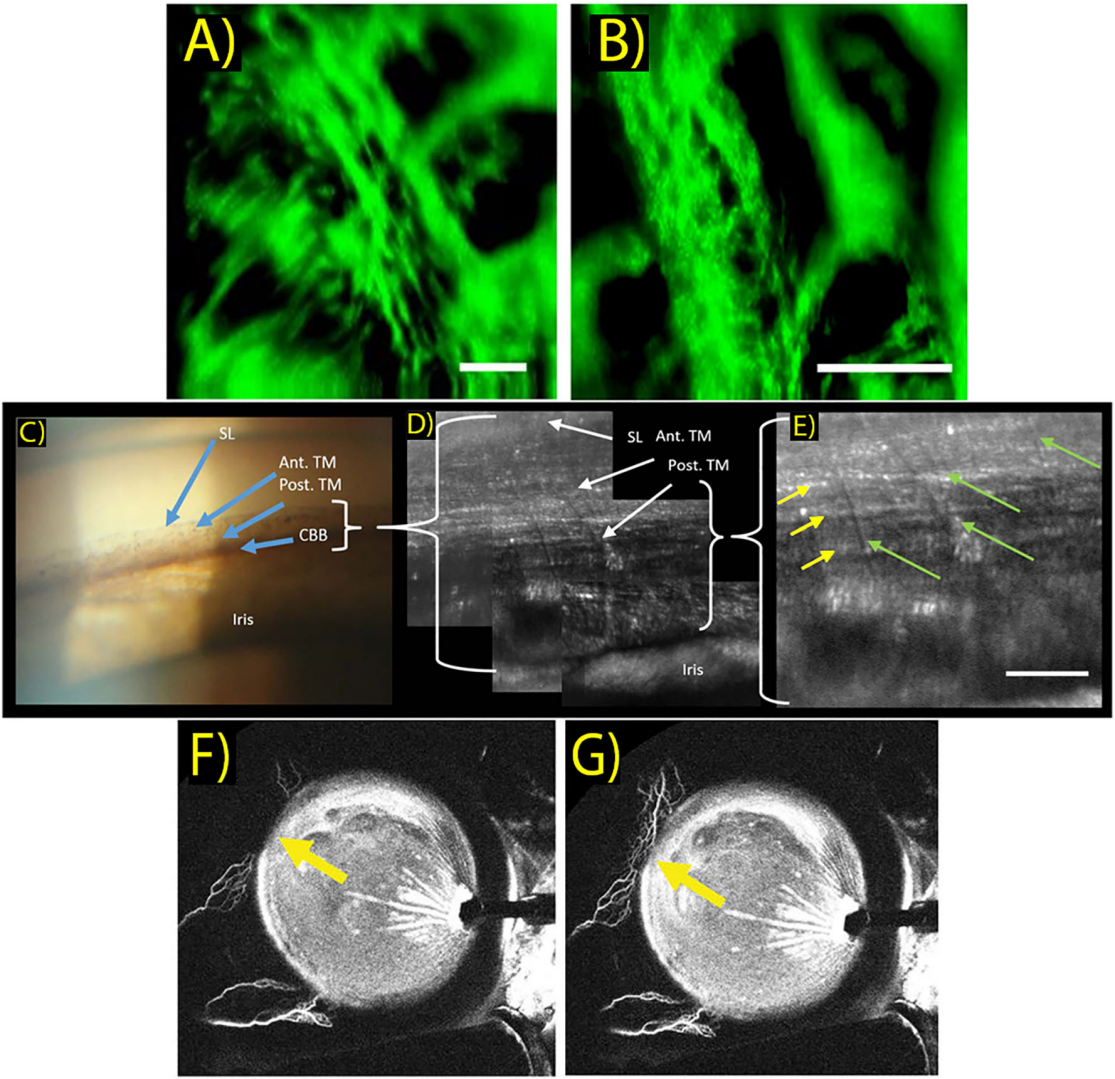
**(A, B)** *In vivo* two-photo images of the trabecular meshwork in two different scanning areas. Images reveal limbal ultra-structure and individual cells within the juxta-canalicular tissue. Scale bar: 50 μm. (Sci Rep. 2019 Dec 1;9(1). Originally published by and used with permission from Nature Publishing Group.). **(C)** Clinical gonioscopy photo using a commercial goniolens and slit lamp camera. Blue arrows indicate Schwalbe’s line (SL), anterior TM (Ant. TM), posterior TM (Post. TM) and ciliary body band (CBB). (Transl Vis Sci Technol. 2019 Sep 1;8(5). Used with permission.). **(D, E)** AOG images of the same iridocorneal region in the same participant. Images acquired with intermediate **(D)** and maximum **(E)** magnifications of the AOG system while focused on the posterior trabecular meshwork. Uveal beams are resolved as shown by the green arrows, while deeper corneoscleral beams are denoted by yellow arrows. Scale bar: 50 μm. (Transl Vis Sci Technol. 2019 Sep 1;8(5). Used with permission.). **(F, G)** Aqueous angiography of aqueous outflow channels performed during cataract surgery with 0.4% indocyanine green diluted in balanced salt solution. Yellow arrows show increased indocyanine green signal of aqueous humor outflow seen in the nasal quadrant between different time points shown at 176 seconds **(F)** and 182 seconds **(G)** after tracer introduction. (Clinical and Experimental Ophthalmology. 2018 March 46(2):158–68. Originally published by Blackwell Publishing and used with permission).

Ex vivo 2P microscopy TM imaging of the last decade was highly valuable for establishing cellular level information regarding TM structure (55–57). More recently, Avila et al. reported a safe *in vivo* technique to non-invasively image the human eye, and showed 2P images of living human TM, sclera, and cornea for the first time (**Figures 2A, B**) (58). Two subjects were imaged with the 2P prototype system that uses a trans-limbal incident beam perpendicular to the TM. Acquired TM images revealed structural and individual cell detail within the juxta-canalicular tissue at a pixel resolution of 1.5 μm. Though the apparent center of interest in their report was on cornea imaging, Avila et al. discuss the potential value of using 2P imaging to track effects of high IOP and characterize TM morphology to aid in diagnosing glaucoma. Non-OCT based imaging techniques discussed in this manuscript are summarized in **Table 2**.

**Table 2:** Summary of discussed non-OCT based imaging techniques.

Technique	ICA Structures Visualized	Spatial Resolution (μm)	*In vivo* human images
Two-photon microscopy	TM, limbus, and juxta-canalicular tissue	1.5	Yes
Bessel beam/axicon	TM networks	2.19	No
Adaptive optics gonioscopy	Schwalbe’s line, corneal collagen, anterior and posterior TM	2.5	Yes
Aqueous angiography	Conventional aqueous humor outflow pathways	Not discussed	Yes

### 4.2 Bessel beam/axicon assisted

Utilizing Bessel beams generated with axicon lenses in ICA imaging provides many advantages, and has been explored to attain high-resolution images in ex vivo and *in vivo* animal models. The ability of Bessel beams to “self-heal” around light scattering obstacles improves tissue penetration while maintaining enhanced contrast to provide intrinsic optical sectional capability. An axicon-assisted gonioscopy approach was described by Perinchey et al, who designed a prototype setup featuring a handheld probe with a central imaging sensor optimized to attain ICA images at different depths (59). Although they demonstrated a 3 μm lateral resolution with this concept, many system difficulties were described in its operation. They only reported images in ex vivo porcine eyes and reported many user difficulties. Hong et al. reported a prototype system also implementing an axicon lens to generate Bessel beam illumination in a light sheet fluorescence microscopy (LSFM) scheme (60, 61). This resulted in a more accessible system with a long working-distance objective, to impart a noncontact nature to image acquisition. This LSFM modality required the application of fluorescein injection to ex vivo porcine tissue and topical drops to the *in vivo* rabbit eye, then imaging from the opposite angle at a 20mm working distance. With this improved imaging platform, TM network arrangement could be visualized with a lateral spatial resolution of 2.19 μm. Although utilization of this imaging technique has not yet been performed in humans, the authors suggest can complement existing imaging methods to help clinicians with glaucoma diagnosis.

### 4.3 Adaptive optics gonioscopy

High resolution imaging of the trabecular meshwork was also recently achieved with a first-of-kind, proof-of-concept paradigm that utilized an adaptive-optics scanning laser ophthalmoscope (AOSLO) to image *in vivo* human trabecular meshwork through a custom modified gonioscopy lens (62). This system employed a button lens centered on the surface above the mirror on a clinical gonio lens. This allowed the AOSLO to focus onto the TM in a gonioscopy approach.

This adaptive optics gonioscopy (AOG) approach proved successful and provided *in vivo* cellular scale (2.5μm) resolution of human ICA structures including Schwalbe’s line, corneal collagen, and posterior and anterior TM structures. (**Figures 2C–E**) While system optimization is required, this setup generated a valuable closer look into meshwork features, as TM endothelial cells and macrophages could be visualized with morphology consistent with findings based on histology. As with any gonioscopy approach, physical contact is also required between the cornea and gonio lens, which may be uncomfortable during the longer imaging sessions described, and the AOG method also prohibits optical sectioning.

### 4.4 Aqueous angiography

Assessment of aqueous humor outflow pathways has also been an attractive area to scientists and researchers interested in exploring aqueous outflow mechanics. This information can be used to develop and optimize interventions to lower IOP, most notably in the form of MIGS. Visualizing conventional aqueous outflow has been established by different aqueous angiography (AA) techniques (**Figures 2F, G**) (63, 64). These strategies, including fluorescein AA and AS-OCT angiography, are very much akin to their respective posterior segment strategies that are already clinically utilized.

Huang et al. were the first to perform fluorescein AA in live human subjects, and have demonstrated segmental, pulsatile, and dynamic aqueous outflow patterns (65). Human AA imaging is performed by tracer addition into the eye, followed by fluorescence capture by an angiographer on a customized arm. An experimental method was developed that sequentially delivers different tracers, which can be used to assess the conventional outflow system before and after intervention. This information can potentially inform future decision making about MIGS placement in the eye. In a recent case study, putative post-surgical recruitment of previously unvisualized aqueous outflow channels was demonstrated in a patient who underwent bent needle ab-interno goniectomy (66, 67).

## 5 Conclusion

Many approaches to obtaining high resolution images of human ICA are still in prototype and proof-of-concept phases. Additionally, while current ICA imaging capability has certainly improved the ability to see *in vivo* angle structures in 3D, there is still much room for improvement to visualize and investigate the pathologic changes in glaucoma. Ongoing research to improve technology and innovation is being conducted in every modality of ICA imaging. Future high-resolution systems will likely incorporate multiple modalities discussed here and utilize AI to help interpret and analyze images. Those systems aiding imaging of the degree of openness of the angle through 360 degrees will help improve predictions of angle closure likelihood and consistency of assessment across the clinician population. With respect to open angle glaucoma, high-resolution ICA research imaging systems should seek to investigate aqueous outflow resistance due to TM pore size and debris accumulation. An improved cellular-level understanding of these regulatory processes and *in vivo* tissue response to interventions like selective laser trabeculoplasty will help aid timing and performance of IOP-lowering procedures. Visualization of these structures in high-resolution can elucidate key biomarkers for open angle glaucoma, like trabecular cell function and detection of morphological changes that correlate with aqueous outflow resistance. Analysis of mechanical stress, flexibility, and the spatial arrangement of collagenous TM beams can possibly provide prognostic information in open angle glaucoma.

New commercial ICA imaging systems will aim to improve physician consistency with categorizing glaucoma and guide decision making for glaucoma interventions like MIGS. Increasing the ease of which the ICA is currently assessed in the clinic is also likely to increase assessment rate and accuracy among clinicians. Application of artificial intelligence to these images should provide helpful assistance to clinical image analysis (68). The next generation of imaging devices seeking to gain high-resolution details of the ICA and its 3D structures will be immensely valuable to vision researchers and clinicians seeking to better understand glaucoma, identify risk of development and progression rate, and deliver more targeted treatment.

## Author contributions

MK: writing – original draft preparation, editing and review; investigation and compilation of research. TG: writing – editing and review. BK: writing – editing and review; investigation and compilation of research. All authors contributed to the article and approved the submitted version.
